# Miscellanies

**Published:** 1835-10-01

**Authors:** 


### MISCELLANIES.

### MADDEN'S RESIDENCE IN THE WEST INDIES.

Dr. Madden is well known to our readers by his various travels in the east—travels which have delighted and improved the reading world in general. The present volume will keep up the interest which Dr. M's former works have excited; and the present field of his observations is still more intimately associated with our feelings and curiosity than the scenes of his former peregrinations among the pyramids, the deserts, and the Arabs of the Eastern world. Dr. M. went to the West Indies in October 1833, as a stipendiary magistrate, and filled that office for twelve months, when he got sick of so irksome an avocation, and returned home. Like a real and practised traveller, he had his eye upon every object, and nothing escaped his pen or pencil. The result is a couple of highly entertaining volumes, full of interest to the philanthropist, and to every one who is anxious for the dignity as well as the happiness of man—both of which are so deeply implicated in the great experiment of Negro-Emancipation, now at length hazarded by this enlightened nation. Even the passage out to Barbadoes affords our author scope for many humorous delineations—but the islands themselves, with their heterogeneous population, black, brown, and fair, afford Dr. Madden inexhaustible supplies of materials for reflection as well as delineation. We think we could contruct an entertaining article out of these volumes, but dare not transgress the boundaries of the *auxiliary sciences* at the utmost—and rarely even the limits of practical medicine. We must exhibit two or three short extracts on medical topics, as specimens of our author's stile and manner. The first is on YELLOW FEVER.

"The accurate description of one virulent pestilence might serve for the general outline of all its forms; and whether the medical observer has to describe the outbreak, progress, and result of yellow fever, of plague, or cholera, he has the same general phenomena to give to the disease; the same indefinite notions of its origin to observe, the same ignorance of its nature to lament; the same conflicting advocacy of its contagious and non-contagious properties to sicken at the sound of; to notice the same leading symptoms of the sudden depression of the vital powers, the same fatal prognosis of the collapse, the same prevailing disorder in the public mind, the same perplexity in medical opinion, the same ignorance of the disease, and in the majority of its attacks, the same event —death.

And as the pestilence happens to embody its miasma in any peculiar form determined by local influence,—an inflammatory fever in the West Indies, a congestive fever in the Levant, or an asthenic one in Hindostan, or where else it may have travelled,—call the disease yellow fever, plague, or cholera, as the poison may be determined to the gastric and cerebral organs, or to the glandular system, or to the mucous membrane of the alimentary canal—the medical observer has to describe a pestilence that originates he knows not how; that first visits the abodes of wretchedness and squalor, and disappears for a season, or diminishes in virulence to return again and expend its fury over the community at large; that is most fatal at its outbreaks, and least so, compared with the number of cases, as it declines; that appears to destroy life by its sudden seizure on the nervous energy; and like the poison of the rattle-snake, though in a less concentrated form, to exert its baneful influence at once on the very principle of life, and to baffle the physician 'who choaks his art' to arrest its progress, and to do with physic what nothing but the regulations of a medical police can accomplish,—to prevent its dissemination by placing its strongholds under the influence of pure air and clean water, by undertaking the direction of the immediate burial of its victims; and lastly, (whatever ridicule may attach to the suggestion of one who has only some acquaintance with each form of the pestilence to oppose to prevalent opinions,) to obviate danger, to avoid disagreeable effects that may arise from causes independently of any specific contagion, by destroying in every instance the bed and bedding of its victims. It behoves me to pull up—I am on the verge of medical politics, and none but fools and tyros rush where doctors fear to tread." Vol. 1, p. 72.

The sangaree system is thus archly satirized.

"The saloon is destitute of windows; but there is no dearth of doors on either side, and these lead to the bed-rooms. Carpets, window-curtains, grates, and hangings, are, very properly, no part of the paraphernalia of the saloon; but, in lieu of these, the stranger slides, at the risk of his neck, over a highly-polished floor; and sits down, as he imagines, at the peril of his life, in a state of liquefaction, in a thorough draft, and for the prevention of cold makes his first call for a glass of sangaree; and in the course of half an hour, to obviate the heat, which is fusing his yet 'too solid flesh,' he is advised to have recourse to the old, simple, unadulterated, 'and best beverage after all'—plain water *diluted* with brandy:—and, before he goes to dinner, to give him an appetite, and dissipate the confounded languor that clogs his energies, he cannot decline a small wine-glass full of bitters mixed with Madeira."

We must close our short notice in this number, with the following poetical sketch of a tropical night, as it is first experienced by a JOHNNY NEWCOME, in either the Eastern or Western hemisphere, within the tropics.

"It is the hour,
When churchyards yawn, and demons lower,
And the confounded roaches-cock
Crawls o'er the bed that's made of flock;
And vile mosquitos pierce the net
That is not made of jaconet;
And horrid ants, in lengthen'd train,
March up your sheets and down again;
When watchful strangers to and fro
Are tossing, and would gladly know
At what degree of Fahrenheit
A man may get a slumber light:
And fain would greet the morning gun,
To drown the buzz his ears doth stun.—
Or hear a Charley cry, 'Move on,'
And tell the world 'Tis half-past one!'
But gas proclaims 'night's cheerless noon,'
To not a 'minion of the noon,'
In Negroland—and one must lie

Awake, in darkness—as did I
Full many a night at that lone hour
When incubi exert their power;
And supper-eaters go to rest,
And dream of mountains on their breast;
And Indigestion has unfurl'd
Her flag o'er half the white-faced world."

In our next number we shall give a few more extracts from these highly amusing volumes of Dr. Madden, still keeping to those parts that are interesting to the medical profession. Mean time we strongly recommend the book to the libraries of our various societies now scattered throughout the kingdom.

---

PHILANTHROPIC ECONOMY, OR THE PHILOSOPHY OF HAPPINESS. By MRS. LOUDON.

Although this literary and political curiosity is not from the pen of a doctor, it is the product of a doctor's wife, and on that account alone, lays claim to a short notice. The subject, too, is not entirely unconnected with medicine. *Economy* is the order of the day, both in and out of the profession—and it is not the worse for being *philanthropic*. In respect to *Philosophy*—she is hand and glove with Physic—witness the four splendid volumes of Dr. Thornton—the "PHILOSOPHY of MEDICINE." HAPPINESS, too, cannot exist without health—the end and object of physic! Indeed there are very few chapters in this talented work of Mrs. Loudon, which might not claim some kindred with the legitimate aim of the healing art. Thus our fair authoress descants on those causes which have a tendency to "starve the community"—and who will deny that *starvation* is injurious to health? Many pages are devoted to the effects of OVER-LABOUR; it is needless to say that this, also, is extremely insalutary. She treats eloquently of the consequences of anxiety, misery, hopelessness,—in short, the whole fruits of oppression—and who will say that these are not noxious to the *individual constitution*, as well as to the constitution of the state? What doctor, then, unless he wish the government to make work for *himself*, can be other than a true reformer? Mrs. Loudon makes sad havoc amongst the corrupt and self-elected corporations. She is, therefore, a strong ally of the reform medical press. Mrs. Loudon is for abolishing "the laws of primogeniture," as injurious monopolies in favour of *first births*. We think our obstetric brethren will agree with her that all births are, or ought to be equal, in point of property and value—and that every second, third, and succeeding child should not be considered as a kind of "after-birth," to be got rid of as speedily as possible, and thrown away as useless to the family. In fine, if Mrs. Loudon's tenets come to be acted on (and they are in the ascendancy at present) we may hope to see the evils of the body politic as effectually purged off by her salutary state physic, as those of the human body are by No. 4 of the springs in her own town of Leamington.

To conclude, we recommend Mrs. Loudon's cheap book to the attention of medical reformers—a numerous and influential class, who can jog the memories of their parliamentary patients, by hints from this comprehensive, yet compendious digest of political knowledge. Mrs. Loudon's principles harmonize very much with those of Mr. Coombe, already noticed in this Journal; and she leaves Miss Martineau in the shade, by the boldness of her views, and the uncompromising character of her political maxims.

---

A SERIES OF ANATOMICAL PLATES IN LITHOGRAPHY, &c. &c. &c. By JONES QUAIN, M.D. &c. &c. &c. Fasciculi XVIII. to XXVI.

These plates are excellently executed. Dr. Quain has now commenced the publication of the plates of the arteries. They bid fair to surpass even their predecessors in accuracy and in beauty. We strongly recommend these plates to anatomical students. We would suggest to Dr. Quain rather to increase the number, in order to render them as copious as Bourgery's. We are sure the purchaser will gladly secure extension of the work, if that will render it more complete.

---

THE HARVEIAN ORATION, June 1835. By SIR HENRY HALFORD, Bart.

The sudden death of Sir George Tuthill left the illustrious HARVEY without an orator—had not the President of the College stepped forth, and, at a very short notice, supplied the place of the deceased socius. It is probable that very few of the Fellows could have so promptly and ably filled up the vacancy as the veteran who accepted the office. This oration is very short; and, curious enough, the name of Harvey does not occur—except in the title-page! We are very far from imputing this as a fault to the distinguished orator. We commend the omission. The orator ought always to be free from any shackles as to the subject of his oration, and under no necessity to introduce the name of Harvey, at all, in the body of the discourse. The present oration contains four concise *eloges* to the memory of four recently departed Fellows of the College—Sir George Tuthill, Dr. Maton, Dr. Ainslie, and Dr. Powell. The characters of these personages, moral and medical, are rapidly, but graphically portrayed. To the memory of Sir George Tuthill a feeling and felicitous tribute is paid. It appears that he carried off the "primi ordines honoris," at Cambridge, and that, in him were centred—"plurimæ literæ, nec eæ vulgares, sed reconditæ." Of valetudinary health, from infancy, he was seized in the spring with laryngitis which proved fatal, after six days of great suffering. Bronchotomy was performed, at the patient's own request—but he died immediately after, or rather before the operation was finished! "Nam peracto vix, ac ne vix quidem opere consumptus est." He is represented, and we think, justly, as exhibiting gravity without severity, cautious and clear judgment—benignity, clemency, and ability. The gravity of Sir George Tuthill's countenance was sometimes so extreme, that he assumed an aspect truly saturnine. There is a well authenticated anecdote of a lunatic who, after staring, for a moment, on Sir George, exclaimed with great emphasis —"get thee behind me SATAN!!" This, however, was one of the many instances, in which physiognomy was at fault. We had some knowledge of the deceased, and, abating the natural prejudices in favour of *his order*, and the equally natural inclination to benefit himself, in the way of his professional pursuits, we believe him to have been a very honorable and upright character.

We are gratified in this place to find Sir Henry Halford discountenancing the practice of instituting a class of *mad doctors*, for the cure of the insane. Really Sir Henry appears half-inclined to be a *leveller!*

"Tollendos igitur *hos limites* esse puto, et philosophiam medicinæ sociam et ministram, adsciscendam:—nec in pharmacopolarum officinis *tantum*, sed in *scholis* quoque sapientûm, quærenda remedia malorum."

In this sentiment we perfectly agree with the illustrious President.

Of Dr. Powell we knew little—of Dr. Ainslie less. The *former* published some papers, and was physician to Bartholomew's Hospital. The *latter* pursued the "noiseless tenor of his way" to the grave—for, during the last 17 years, we hardly knew that such a man existed amongst us, except by the name on the door in Dover-street.

Of Dr. Maton, Sir Henry has drawn a just character. From local or accidental circumstances, it happened that we had a more intimate *professional* acquaintance with Dr. Maton than with any other Fellow of the College. Though we were opposed to each other in medical politics—and, during the late fatal epidemic, in medical doctrines also, yet we always found Dr. Maton to be a man of strict honour, of mild demeanour, of rigid punctuality—in short, a practiser of the most sound medical ethics. Nothing could be more true than the following characteristic of Dr. Maton, in language resembling that of our Saviour.

"Nam quod decens et honestum esset facilè percipiebat, nec alteri faciebat unquam quod sibi faciendum noluisset."

We have thus discharged a duty—a grateful one—to a political opponent, but a private friend! To Dr. Maton we would have entrusted our personal property—but not our public institutions. The *former* would have been perfectly safe in his honest and honourable hands :—the *latter* would have been endangered by that bias of *judgment*, which is generally inseparable from education in particular tenets.

We were struck with the following passage.

"Me, si deo placuerit ingraviscentibus annis, imbecilletate valetudinis impediteri—esto. Id omne benevolentiæ ejus acceptum referam. Sin otii fructus detur, cum sana mente in corpore sano, ad vitam ante-actam recogitandam, et vestem, quasi colligendum, quemadmodum Imperatori ante cadendum in Capitolio curæ fuit, ut decorè, et cum dignitate discederet, laudem Deo majorem, majoresque gratias debiturus solvam."

Sir Henry Halford has lost the opportunity of winding up a long and prosperous professional life, with additional honours to himself, and benefit to his brethren, by using his influence in liberalizing the Institution over which he presides, *and* thus making it the bulwark and pride of the medical profession in this country, instead of a mark at which the arrows of scorn, ridicule, or hatred, have been daily levelled. We long had hopes that the keen-eyed president would have foreseen the policy of liberal measures, before it was too late. The announcement of a metropolitan university of "ONE FACULTY," after all—must prove the funeral dirge of the College!

We apprehend that very few, after the establishment of a Metropolitan University, will gratuitously throw away fifty or sixty pounds for the honour of a license from the College of Physicians, which can be of no use whatever to them in practice. It was only the other day that one of the most talented and influential Fellows of the College declared publicly, in the hearing of many professional men, that "THE COLLEGE WAS EFFETE," and might close up its doors, for there would soon be none to enter them!

---

### DISEASES OF THE SKIN. ATLAS OF PLATES.

It was our intention, as will appear from our review of Rayer and Green's works in the present number to have selected some of the most instructive cases therein detailed, with the view of illustrating the very important subject of cutaneous diseases.

The great press, however, of materiel of a most interesting and useful description, which we have collected from numerous sources during the past quarter, has prevented us from doing so. This omission we regret the less, as our readers will very probably be induced by the recommendations which we have worthily bestowed on the treatises of Drs. Rayer and Green, to examine them attentively for themselves. Indeed no well-informed physician of the present day can dispense with a most diligent perusal of the great and excellent work of the French author; and to the younger student of medicine we do not hesitate to recommend the compendium of Dr. Green in preference to Bateman's Synopsis, (no mean praise,) as more simple in its descriptions, and more practically useful in its therapeutic instructions. It is unnecessary to say more of either of these works.

The study of dermatology, as of botany, is greatly facilitated by a series of well-executed drawings.

The Atlas, which accompanies Rayer's Treatise, consists of twenty-two plates, each containing from twelve to eighteen figures, in which are represented most accurately almost every species of cutaneous disease.

With the exception of very few, the figures were drawn from the living subject by one of our own countrymen, Mr. James Young, of Paisley, who has proved himself a most admirable draughtsman. Certainly we have never seen any drawings of disease from the foreign press equal to those contained in this Atlas. They have little of that peculiar artificial-like mannerism which almost inevitably marks the productions of the French graver. Four of the plates are devoted to the illustration of the Protean forms of syphilitic eruption, and on the whole they convey a very faithful representation of their more obvious and common character. The value of the Atlas is greatly enhanced by its thus affording to the less experienced practitioner a standard of reference, to guide him in the discrimination of this most varying and multiform disease.

---

BANFFSHIRE MED. CHIRURG. SOCIETY.

*To Dr. James Johnson.*

Sir,

I am instructed to forward to you the following minute of the proceedings of the "Banffshire Medico-Chirurgical Society."

"The Banffshire Medico-Chirurgical Society, taking into consideration your deservedly prominent rank in the medical profession, as well as the ability and industry which you have displayed in the cultivation of science, more especially of such branches as tend to the advancement of medicine, have, as an expression of their admiration of your talents, elected you as an honorary member of that society with all the privileges; and have instructed their secretary to transmit this notification of the same."

I am also directed to intimate that this society was instituted in 1834, for the purposes of forming a library, museum, &c. &c., and of communicating all interesting cases which may happen in the course of practice, of reading papers on subjects connected with medicine, and of endeavouring to keep pace with the advancing state of medical literature throughout the kingdom.

I have the honor to be,
Sir,
Your obedient Servant,
HENRY MILNE, Sec.

*Banff*, 16*th July*, 1835.

### Bibliographical Obituary;

OR THE

LIFE AND DEATH OF THE MEDICAL QUARTERLY REVIEW, WITH THE APPEARANCES ON DISSECTION.

It was only in January of last year (1834) that we were called upon to deliver a funeral oration at the grave of our oldest contemporary—the MEDICAL and PHYSICAL JOURNAL—the last of the MOHICANS, or at least of the MONTHLIES! Little did we suppose that we should so soon be summoned to perform the same obsequies at the tomb of its son and successor. There certainly were some inauspicious circumstances attendant on his birth.—Young MASTER QUARTERLY was delivered by a kind of Cæsarean or *post-mortem* operation, some time after the decease of his parent; who, having been, for many years, completely *jaundiced,* sent the baby into the world with "icterus infantum," or "yellow gum," from which the young QUARTERLY was never able to clear its skin, during the brief span of its existence. The youngster, if not a child of great *hope,* in the eyes of others, was certainly of much *promise* on his own part. Being born in a church-yard, he evinced a strong predilection for the *dead languages*—and was so profoundly and precociously versed in *tongues,* that one would have supposed he had been born on the tower of Babel. He could quote Arabic, Hebrew, Hindoo, Latin—aye and—

——————— Greek,

As naturally as pigs squeak.

He was a profound medical politician, from his cradle. Being brought up at the very foot of Elizabeth's statue, he was a great stickler for the "monarchical principle" in physic; and therefore he worshipped, even to idolatry, the gods and demigods of PALL-MALL EAST. It was clear, indeed, that MASTER QUARTERLY had an eye to the FELLOWSHIP, from the moment he was capable of distinguishing light from darkness. The daily contemplation of ST. PAUL's probably led our young contemporary to be a "HIGH CHURCHMAN," in medical politics. In his eyes,

Oxford and Cambridge were the only places to acquire professional knowledge, as well as morality and religion. Subscription to the 39 articles, on bended knee, in Pall-Mall East, he considered as the only criterion of medical science and of fine " moral feeling." All below the FELLOWSHIP, or beyond its pale, were illiterate licentiates or rank radicals, in his estimation. An ardent admirer of our "venerable institutions," the young QUARTERLY, " hated the very name of reform" in physic, as heartily as Lord Stormont did in politics.

Being cut off within the first two years, his teeth were very little developed, and his *bites* were never considered as very formidable or dangerous. His " dentes *sapientiæ*" never made their appearance, of course—and few even of his *grinders*. In his *critical* avocations, however, he made good use of his *incisors*, and not unfrequently drew blood from the fingers of those who were soft enough to examine the young lion's mouth. He experienced very few of the infantile or eruptive diseases, except the WHOOPING COUGH, of which he had a violent attack once in every three months. In each of these paroxysms, great alarm was entertained by the friends of the young prodigy. The *inspirations* were observed to be stridulous, and far from *filling the chest*; while the expectoration was of a *frothy* and unsatisfactory kind. The " râle" was sometimes " sibilant"—sometimes " crepitant"—but the breathing was always laborious, and the *circulation* confined. The attendant physicians apprehended *hydrothorax*, but Dr. Webster was positive that the source of the malady was in the *head*. In the eighth paroxysm, about the end of June last, the patient was seized with LOCK JAW —a symptom which had never been anticipated—and departed this world on the first day of July—aged one year and nine months!

Abstulit clarum cita mors ACHILLEM!

Having no landed or funded *property* to bequeath, the deceased left his MANTLE and his BLESSING—not to any of his poor *friends*—(some of whom were not out of the need of such a God-send)—but, to a *fœtus in utero*—of which its mother, Mrs. Forbes, of Chichester, declared herself *enceinte*, and three months gone. Mr. Connolly, of Warwick, the father, deposed to the truth of this statement. Although the embryo had not even *quickened*, at the time of the testator's death, yet the latter, in one of those prophetic extacies, which Sir Henry Halford has so often witnessed at the dropping of the last curtain, declared that the forthcoming stranger would prove a second CRICHTON—and that, through his influence, a *physical* revolution would be wrought in the *periodical* world.

Many conjectures were hazarded as to this bequest of the mantle and benediction to a BEING yet unborn; but the enigma was solved when it appeared that a noted midwife, of the name of ADLARD, residing in Bartholomew Close, and who had helped the DAM of the dying kid through many a lingering labour and perilous abortion, was the " SAGE FEMME," engaged by Mrs. Forbes to deliver her of her precious babe on the first of January next, 1836.

### AUTOPSY.

It was not deemed necessary to examine, with much minuteness, any other *cavities* than the *head* and the *chest*. In the latter, *nothing* was found. The stomach contained some *undigested* matters which, on examination, proved to be half-masticated leaves of old books and illegible manuscripts. In the bladder were found a good deal of *gravel*, which the deceased had been in the habit of discharging periodically, with considerable *difficulty*. It consisted chiefly of the triple salts—the *attic* being very conspicuous. The cystic duct was obstructed by a biliary concretion, which accounted for the colour of the skin, and the want of *gall*, latterly, in the *egesta* of the deceased.

But the head presented several remarkable prominences, and Drs. Elliotson and Epps were requested to examine it with their callipers. They did so accordingly.

The organs of AMATIVENESS and PHILOPROGENITIVENESS were not developed, of course, in so young a subject, and therefore it is a matter of conjecture whether or not there would have been issue, had the young "TWO-YEAR-OLD" arrived at maturity.

*Language.* This organ was "prodigious." Our phrenological readers are aware that the organ of language lies directly above and behind the eye, resting on the orbitar process. That process was bent down under the pressure of the organ, so that the eye required powerful lenses to prevent erroneous impressions on the retina.

*Veneration* was at least double its usual size—hence the worship of the *magnates* in Pall Mall East.

*Locality* was dissimilar and even divergent on the two sides of the brain.

*Time*, very short.

*Number*, very small.

*Tune*, Saul's march.

*Weight*, very light.

*Concentrativeness*, not remarkable.

*Self-esteem*, enormous! This organ is situated at the very top of the head, and is placed between two others, the "love of *approbation*." The trio had grown to such a magnitude, that several neighbouring organs were annihilated entirely! Neither Dr. Elliotson nor Dr. Epps could find any trace of *constructiveness, acquisitiveness*, or ALIMENTIVENESS. This *last* circumstance, coupled with the well known fact that, even the greatest genius cannot now live upon APPROBATION alone, may account for the premature death of our juvenile contemporary.

In other respects, there was nothing very extraordinary in the head. The cranium was rather thicker and harder than is usually observed in such young subjects; and the brain, in certain parts, had undergone some degree of *ramollissement*.

There is no case in medicine from which some useful information may not be drawn. In the foregoing instance, we observe that, although the organ of "SELF-ESTEEM," or rather the propensity of which it is the representative, is a very useful organ, if within proper bounds, yet when overgrown, it leads to variety, conceit—and, what is worse, to contempt of our neighbours. "Love of approbation," if moderate, prompts to useful or even noble exertions—but if inordinate, it generates boundless ambition, or love of fame, which heeds not the misery or inconvenience of others, if they stand in our way! An inordinate addiction to *language*—to words rather than things—too often begets a propensity to *pedantry*, than which there is nothing more insufferable.

But we should not have given way, even to this harmless and jocular allegory, had not our deceased contemporary, in various parts of his short career, levelled pretty significant sneers at his fellow-labourers in the vineyard of periodical literature—a practice by no means uncommon among neophyte journalists, and one which we regret to see recently adopted in a quarter where we little expected such a procedure. We ask those who are about to undertake the laborious and the *hazardous* task of originating and sustaining a new medical journal, whether it is very *prudent* to preface their intentions by such a passage as the following?

"There seems the fullest reason to believe, notwithstanding the *great and acknowledged ability* displayed in the medical periodical press, that, in the actual state of medical literature, a work of *higher critical aims* than are professed by the conductors of the *principal* journals in this country, is really desired by the members of the medical profession."

Is it wise—is it *modest*, after such a statement, to announce themselves as the editors who are about to rise above this "acknowledged ability," already in the market, and take *higher* grounds than any even *profess* to aim at, in the year 1835? This is quite analogous to the language held, two short years ago, by the defunct Review; and, indeed, it is the usual prelude to new journalism in general. Yet, in all enterprizes attended with danger of failure, it is surely most *impolitic* (to say the least of it) to lay claim to merits or talents superior to those already in operation, thereby directly insulting the whole of their competitors. This self-exaltation will do them no credit, even if they succeed in driving the whole of their rivals out of the field; but, should miscarriage take place—an event to which all are liable—see, then, to what ridicule they have justly exposed themselves by this gratuitous and wanton wound inflicted on the feelings of their contemporaries. In this respect, we can look back, without shame or remorse, to the outset of our own labours, and we think our readers will pardon us for quoting the following passage from the preface to the first volume of the quarterly series of this Journal, June 1820.

"As one of the now numerous candidates for performing this office, the Medico-Chirurgical Review has presented itself before the profession, in its present form, without any wish to depreciate its cotemporaries, or to magnify the necessity that existed for new labourers in the vineyard of periodical literature. Such an attempt would be equally impolitic and illiberal. The PUBLIC is a stern, inflexible, and, finally, unerring tribunal, before which it is unnecessary to plead the cause of merit, and useless to varnish infirmity.—Friends may flatter, and enemies may defame, but the Public at large do justice, because they are far removed from the sphere of personal feeling and influence. It is to this tribunal—and to this tribunal *alone*, that we shall ever appeal."

To these sentiments we still adhere, after fifteen years of further experience.

The Editors of the Journal announced dwell, with great satisfaction, on the advantages of "free and candid critical discussion"—and also on the "just source of complaint, on the part of authors, that this important task *has been confided to persons little qualified to undertake it.*" Here, then, we have a general and sweeping denunciation against the whole of the medical periodical press of this country! Let us be permitted to quote a passage bearing on this subject, from the first volume of this Journal, already referred to.

"There is, however, a class of medical society, numerically not great, but potentially important, on which this Journal may, probably, have some claims—it is the class of MEDICAL AUTHORS. To analytically portray their works before the whole professional circle, leaving the judgment in general, to the Public itself, is no mean service to authors—no common advantage to readers—no sinecure office for reviewers—no inconsiderable check on hypercritics.—Whether the MEDICO-CHIRURGICAL REVIEW executes this task, with any degree of credit to itself, or satisfaction to the Public, it is for that Public to decide. If it does—their patronage will, in justice, be continued—if it does not, it will, with equal justice, be withdrawn."

To this principle we still adhere—and we boldly and fearlessly place it in juxta-position with that principle which is set forth as its superior. Fifteen years have enabled the public to decide on the comparative merits of analysis and criticism—but if that period has not been sufficient, why let them take fifteen years more. In the course of that time, we have no doubt that the new journalists will have ascertained the relative value of the two systems, and shaped their course accordingly.

The fact is, that in no department or pursuit of human knowlege is there less room for high-flown criticism and erudite disquisitions, than in MEDICINE. Practitioners have wisely exchanged speculation for observation—and theory for experiment. Who now puzzles his brains about the doctrines of Van Helmont, Stahl, Boerhaave, Cullen, Darwin, or Brown? The utility of medical *criticism* consists chiefly in the ability (acquired by observation) to distinguish truth from error—and facts from fictions. This power of discrimination needs no *critical disquisitions*. It is exexercised, and exercised best, in few words. But the great labour of the journalist, after all, will be *analysis and concentration*. The deceased journal did not think so. It set off with high pressure steam on the sea of CRITICISM; but criticism was the rock on which it split—and is the grave in which it lies buried. When it had plenty of sea-room and fine weather, it steered directly for the land of promise. When it found itself among the breakers, it tried to tack ship, and haul off into the very course which it first despised. But it was too late. Rudder and sails were useless—it drifted among the shoals—and was shattered against the rocks.

